# Low prevalence of ideal levels in cardiovascular behavior metrics among Mexican adolescents

**DOI:** 10.1186/s12889-023-15959-3

**Published:** 2023-06-12

**Authors:** Ricardo Terminel-Zaragoza, Mariana Angulo-Urías, Iván de Jesús Toledo-Domínguez, Hebert Quintero-Portillo, Cecilia Ivonne Bojórquez-Díaz, Gabriela Ulloa-Mercado, Pablo Gortares-Moroyoqui, Mayra Arias-Gastélum, Fátima Legarreta-Muela, Ana Rentería-Mexía

**Affiliations:** 1 Maestría en Ciencias en Recursos Naturales, Instituto Tecnológico de Sonora, Ciudad Obregón, Sonora, México; 2 Licenciatura en Tecnología de Alimentos, Instituto Tecnológico de Sonora, Ciudad Obregón, Sonora, México; 3 Departamento de Sociocultural, Instituto Tecnológico de Sonora, Ciudad Obregón, Sonora México; 4 Departamento de Unidad Navojoa, Instituto Tecnológico de Sonora, Navojoa, Sonora México; 5 Departamento de Biotecnología y Ciencias Alimentarias, Instituto Tecnológico de Sonora, Ciudad Obregón, Sonora, México; 6 Facultad de Ciencias de la Nutrición y Gastronomía, Universidad Autónoma de Sinaloa, Culiacán, Sinaloa, México

**Keywords:** Cardiovascular health metrics, Mexican adolescents, AHA dietary targets, AHA metrics, College students

## Abstract

**Background:**

Lifestyle changes when transitioning from high-school to college expose students to unhealthy behaviors associated with high cardiovascular risk. The study aimed to assess the cardiovascular behavior metrics according to the AHA criteria, in freshman college adolescents from Northwest Mexico.

**Methods:**

The study was cross-sectional. Demographics and health history were collected by questionnaires. Four behaviors were evaluated: diet quality using a duplicated FFQ, physical activity (PA) using the IPAQ, smoking, and body mass index (BMI) percentile; blood pressure was measured as a biological metric. Intakes were averaged and summed for each food group; sodium and saturated fat were calculated using the Mexican System of Food Equivalents or the USDA Database. Metrics were categorized into ideal, intermediate or poor level according to the AHA criteria. Diet outliers (± 3 SD) were trimmed and data was tested for normality. Mean±SD were calculated for continuous and percentages for categorical variables. Chi-square test compared the prevalence of demographic variables and levels of each cardiovascular metric by sex. Independent T-test evaluated differences in anthropometrics, dietary, and PA by sex, and the prevalence of ideal vs. non-ideal dietary intakes.

**Results:**

Participants were n = 228, 55.6% men, age = 18.5±0.4 y. A higher prevalence of men indicated working, playing sports, and family history hypertriglyceridemia (p < 0.05). Men showed higher weight, height, BMI, waist, blood pressure, and lower PA and body fat (p < 0.05). Concerning diet quality, significant differences by sex were observed in nuts and seeds (1.1±0.6 and 0.9±0.6 oz/week, p = 0.042) and processed meats (749.8±639 and 503.6±300.3 g/week, p = 0.002); only fish and shellfish group reached AHA recommendations (513.1 ± 450.7 vs. 501.7 ± 428 g/week, p = 0.671) for men and women, respectively. Ideal level was reached by 70.9% participants for BMI percentile, 87% for smoking, 67.2% for blood pressure, 25.9% for PA, and 12.2% for diet score. Regarding food groups and nutrients, the lower prevalence in the ideal level was for sugar-sweetened beverages (10%, p = 0.013) and processed meats (4.8%, p = 0.208), and the highest for fish and shellfish (87.8%, p = 0.281) .

**Conclusions:**

The diet and PA patterns of Northwest Mexican freshman adolescents make them a high-risk group for developing long-term unhealthy habits and cardiovascular complications early in adulthood.

**Supplementary Information:**

The online version contains supplementary material available at 10.1186/s12889-023-15959-3.

## Background

During the last decades, cardiovascular diseases (CVD) have been the leading cause of morbidity and mortality worldwide [1]. Actually, CVD deserve special attention during the current COVID-19 pandemic, given that the previous presence of cardiovascular (CV) comorbidities has been associated with high mortality from COVID-19 complications in infected patients [2]. There are multiple risk factors for CVD; among them, obesity, insulin resistance, and hyperglycemia are biological, while unhealthy diets and sedentary lifestyles are behavioral. CVD have their antecedents early in life, through a condition known as atherosclerosis [3]. CVD symptoms usually take 20 years or more to show; however, it is well known that atherosclerosis can develop since the first childhood stages [4], especially in a detrimental lifestyle. Unhealthy lifestyles, such as poor diets and physical inactivity, promote inflammation and oxidative stress through different biological, molecular, and physiological mechanisms, consequently increasing the CVD risk [5]. In this sense, adolescents are a target group for preventing CVD.

According to the American Heart Association (AHA), a new concept to promote the reduction of deaths from CVD and stroke was first introduced in 2010: cardiovascular health (CVH). The CVH definition includes four behaviors: diet quality, physical activity (PA), smoking, and body mass index (BMI); and three blood factors: total cholesterol, blood pressure, and glucose, with a total of seven components. CVH can be represented using categorical ranges for ideal, intermediate, or poor for each of these components [6]. When present at unhealthy levels, both behaviors and biological factors contribute to and exacerbate the atherosclerotic process in the youth [3].

Latinos are disproportionately impacted by obesity and type 2 diabetes (T2D), both of which lend to a worsening CVD profile into adulthood [7]. In México, CVD and diabetes were reported as main causes of death in 2016, with CVD representing 24%, and diabetes 15% [8]. With respect to young age groups (12–19 y), prevalence of overweight and obesity in Mexican adolescents increased from 34.9% in 2012 to 42.9% in 2021, with 24.7% of overweight and 18.2% of obesity. Comparing the prevalence of overweight and obesity throughout the previous nutrition surveys with the most recent in 2021, an upward trend is observed in the obesity category, mainly in adolescent men [9]. When analyzing the obesity prevalence by region of residence, in the 2021 nutrition survey the highest prevalence of obesity was for the North-Pacific (32%), region to which the state of Sonora belongs; in this same region the adolescent men had the highest prevalence of obesity (43.5%). Despite the increased CVD risk associated with overweight and obesity, little attention has been paid recently for assessing lifestyle factors such as PA, smoking, blood pressure, and diet in Northern adolescents [10, 11].

First-year college students or “freshmen” are adolescents in a transition phase with frequent changes in their lifestyles. During this period, it is usual for adolescents to stop eating homemade food and eat in food establishments close to the school [12]. This change in dietary habits allows adolescents to be exposed to unhealthy food products. Self-service establishments and internal stores in schools rarely offer healthy food options, such as fresh fruits and vegetables, low-fat products, good-quality protein foods, etc. As a result, college students have a high intake of processed foods, with high content of saturated fat, salt, and sugar [13]. Furthermore, new academic and social activities for freshmen are time-consuming and often result in decreased PA levels. If these habits continue throughout adolescence, healthy habits would be more difficult to adopt later in adulthood [7]. Some studies have addressed CV risk in university students from different countries with the same worrisome results regarding unhealthy lifestyle habits [14, 15].

Despite the high CV risk posed to adolescents, there is a lack of data referring to the awareness of the AHA components of CVH among Northern Mexican adolescents. Up to our knowledge no studies have documented the CV risk in Mexican students specifically during transition from high school to college, according to the AHA behavior criteria. In addition, Sonora is a state with a great influence from the Western lifestyle, with substantial dietary changes on its population. Consequently, adolescents from Northwestern Mexico have adopted Western dietary habits that are not culturally typical of México. Therefore, it is essential to evaluate the suitability of behavior metrics that constitute CVH in this high-risk population. The present study aimed to assess the behavior metrics related to CVH and the prevalence of ideal, intermediate, and poor levels of these metrics according to the AHA criteria in Northwest Mexican freshman college adolescents.

## Methods

### Study design and ethics

This was a cross-sectional observational study. All data collection procedures were completed at the Laboratory of Preventive Nutrition and Healthy Eating. The Institutional Committee of Ethics in Research of the Technological Institute of Sonora reviewed and granted authorization for the current study to be carried out, including protocol and materials.

### Settings and recruitment

This study was carried out at the Instituto Tecnológico de Sonora (Technological Institute of Sonora, ITSON by its acronym in Spanish), in Cd. Obregón, Sonora, México. Recruitment was done on three occasions in the semester from August to December 2018: 1) during the University Induction Program (before classes began); 2) immediately after classes began; 3) during the semester. Students were approached in this way because their behavior can vary throughout the semester. We recruited students at the university location on each occasion explaining the study rationale, aim, and procedures. Students were invited to participate, and for those interested, a written study summary was given and asked them to deliver it to their parents. Written informed consent from parents and written assent from adolescents were obtained. As an incentive, and in agreement with the university staff, each student who completed the study would receive credits in the ‘’Healthy Lifestyle University Program’’ (each student must enroll in such program during their career). Additionally, during the semester, we got support from career directors who allowed us to visit classrooms to invite students to the study.

### Study participants

Participants volunteered for the present study. Participants were enrolled in the study if they were freshmen from 17 to 20 years of age and willing to complete all the study protocols: questionnaires and behavior measurements by duplicate (dietary data) and triplicate (anthropometrics). Exclusion criteria comprised pre-diagnosed cardiometabolic diseases such as CVD, diabetes, chronic renal failure, and non-alcoholic fatty liver disease, as well as mental illness such as neurocognitive disorders, neurodevelopmental disorders, psychoses, emotional disorders, and externalizing disorders, as all these conditions comprise a specific dietary-, exercise- and/or lifestyle-treatment/therapy that would not reflect the common habits of such participants. Other exclusion criteria were currently taking any medication, pregnancy or breastfeeding, or some other conditions affecting their ability to participate. Participants were scheduled via cellphone calls or text messages, reminding them of the importance of the study for their health and giving the credits as an incentive. Estimated sample sizes were calculated with 95% level of confidence, 0.05 precision and independently using the reported national prevalence of 14% for obesity and 22% for overweight in Mexican adolescents from 12-19y in the National Health and Nutrition Survey 2016 (the most recent prevalence at the moment of the study), then both were averaged resulting in calculated sample size of n = 215. At the time of conducting the study, we tried to recruit as much participants as we could considering the nature of our study design and limitans as financial and staff resources.

### Anthropometric measurements

Participants arrived at the Laboratory following an overnight fast (> 10 h). Weight and fat percent were measured with lightweight clothing without shoes and recorded to the nearest 0.1 kg using a TANITA BC-533 full-body digital scale with integrated electrical bioimpedance. Standing height (without shoes, neither hair devices nor high hairstyles) was measured to the nearest 0.1 cm using a SECA stadiometer. BMI was calculated as weight divided by height squared, used to calculate BMI percentiles based upon the CDC 2000 growth charts for age and gender [16]. Waist circumference was measured after determining the midpoint between the lowest rib and the superior border of the iliac crest on the right side, and registered to the nearest 0.1 cm using a Lufkin tape. For height and waist circumference, the average of three measures was used. All measurements were taken by standardized staff.

### Demographics data collection

Demographics data, family health history, and smoking were collected through questionnaires administered by standardized staff. The socioeconomic level (in US dollars per year) was determined according to the classification established in the 2016 National Survey of Household Income and Expenditure (ENIGH 2016) in Mexico.

### Blood pressure measurement

Blood pressure was measured by standardized staff in a relaxed, upright sitting position using an appropriately sized cuff on the right arm after the participant had rested quietly for 5 min using an Omron baumanometer (Omron Healthcare, Inc., Bannockburn, IL). Measurements were taken at 1-minute intervals, and the average of three measures was used.

### Physical activity estimation

PA was estimated using the International Physical Activity Questionnaire (IPAQ), which was previously validated in Spanish population [17]. Questions referred to the intensity (type of PA), duration (minutes/week), and frequency of the activity (days/week) that participants engaged in during the previous week. Activities were described for participants as vigorous (those that involve intense physical effort and make the participant breathe much more intensely than usual), moderate (those that require a moderate physical effort that makes the participant breathe somewhat more intensely than usual), and light (includes walking at work or home, getting from one place to another, or any other walk participant might do just for transportation or recreation). The questionnaire also asked respondents about time spent in sedentary activities such as sitting. Self-responses were used to estimate the minutes/d participants engaged in each activity and to calculate metabolic equivalent of task (METs/week).

### Food frequency questionnaire (FFQ)

Participants were asked to respond to a paper-based semi-quantitative FFQ adapted from Block et al. (1986) [18] and validated in Northwest adolescents [19]. The FFQ focused on a list of 152 individual foods related to CVD based on the following AHA criteria for food groups and nutrients: fruits and vegetables (F&V); fish and shellfish; sodium; sugar-sweetened beverages (SSBs); whole grains; nuts, seeds, and legumes; processed meat; and saturated fat [6]. Adaptations included regional foods usually consumed in Northwest México [20], with medium portion sizes adapted according to the Mexican System of Food Equivalents [21]. For regional foods not included in the Mexican System, the medium portion was based on weight (either raw or cooked, depending on the usual food intake). Small and large portions were established according to 0.5 and 1.5 medium sizes, respectively. The frequency was set according to the following periods: never, daily, per week (up to 7 d), month (up to 30 d), or year in case of seasonal foods (up to 365 d), with blank space on each option to write the number of times each item was consumed within each selected period.

The FFQ was administered on two occasions (duplicate) over three months in person, in a pen and paper version. Participants were instructed to indicate the number of times each food is consumed and to cross mark their usually consumed portion size (small, medium, or large), according to visual plastic food models (Nasco, Fort Atkinson, WI) and photo-aids (Vitamex Nutrition, Jalisco, México), to help assess portion size. FFQs responses were checked for completeness or missing values and data was double-checked and captured in a Microsoft Excel spreadsheet (Microsoft Co., Redmond, WA, USA). Equations using portion size and frequency consumption were used to calculate either daily or weekly intake from individual foods. Data from individual food intakes were averaged (from FFQ1 and FFQ2) and then summed for each food and nutrient group. For F&V (cups), sodium (mg), whole grains (oz), and saturated fat (g and %) data were calculated by day; for fish and shellfish (oz), SSBs (oz), nuts and seeds (oz), legumes (cups), and processed meat (g), data were calculated by week, according to the AHA recommended intake. For calculating nutrient amounts, data from the two FFQs were captured in a free web app (Nutre.in) using the Mexican System of Food Equivalents, and those not found were checked in the USDA Global Branded Food Products Database [22]; saturated fat was adjusted dividing kcal from saturated fat by the total kcal intake in a day, and multiplied by 100. All data were averaged and outliers, defined by ± 3 standard deviation (SD), were trimmed from each food group and nutrients.

### Cardiovascular metrics and score

Each metric was registered for each screening participant as “ideal”, “intermediate” or “poor”, according to the criteria established by the AHA’s definition for CVH (see Additional file 1) [6, 23–25]. In the case of AHA dietary targets, for better consistency with other dietary pattern scores, the AHA scoring system uses an alternative continuous scoring system. Each dietary target is assessed by use of a range of 1 to 10, with the highest score (10) given for meeting or exceeding the AHA target for protective factors, and for not exceeding the limit for harmful factors (e.g., at least 4.5 cups of fruit and vegetables per day; no more than 1500 mg/d of sodium), and the lowest score (0) given for zero intake (protective factors) or for very high intake (harmful factors). The score for each metric was scaled continuously and proportionally calculated within this range. For harmful factors, the level of high intake that corresponded to a zero score was identified as approximately the 90th percentile distribution of US population intake [25] and Latino adolescents [23] (see Additional file 2). The AHA scoring system sums all components, thus the natural range of the primary AHA diet score is 0 to 50 (sum of 5 components), and the natural range of the secondary AHA Diet Score is 0 to 80 (sum of 8 components). Both scores are then rescaled to a range of 0 (worst) to 100 (best) for comparison purposes. Ideal level was categorized for a score of 80–100; intermediate for a score of 40–79, and poor < 40.

### Statistical analysis

Normal distribution of data was verified according to the symmetry, kurtosis, and the Kolmogorov-Smirnov tests. Statistical and graphical assessment methods were used to determine the most appropriate transformation to achieve normal distribution for BMI and BMI percentile. For normality accomplishment, inverse transformation was applied for BMI, and square-root transformation were applied for BMI percentile and intakes of fish and shellfish, sodium, nuts and seeds, legumes, and processed meats. Means and standard deviations (SD) were calculated for continuous, and percentages for categorical variables. The Chi-square test was used to compare the prevalence of demographic variables and levels of each CV metric by sex. The T-test for independent samples was used to evaluate the differences in anthropometric, dietary, and PA variables by sex, and for comparing prevalence of ideal vs. non-ideal (intermediate + poor level) dietary intakes.

## Results

### Demographic and anthropometric characteristics

**Fig. 1:**
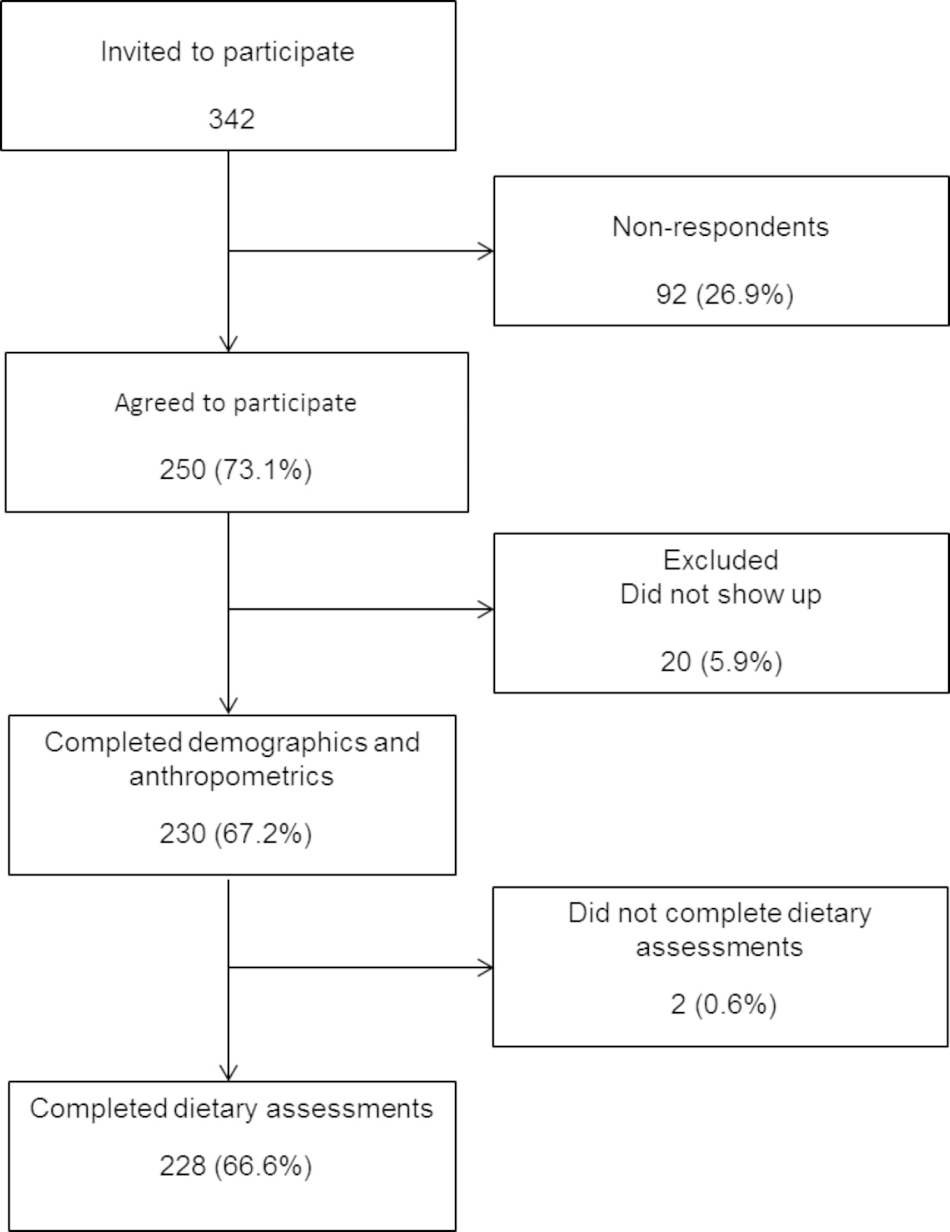
Flow chart of the study participants

A total of 342 college freshman students were invited to participate. From these, 250 (73.1%) students were recruited, and finally, 230 (67.2%) completed family history and anthropometrics, and 228 (66.6%) completed dietary measurements (Fig. 1). Participants had a mean age of 18.5±0.4 y old and were predominantly men (55.6%). Table 1 shows demographic data with no significant differences by sex in age, socioeconomic level, most of the diseases from family history, and smoking (p > 0.05). Significant differences with higher prevalence in men were observed for those who work (p = 0.002), practice sports (p = 0.001), and have a family history of hypertriglyceridemia (p = 0.001). Table 2 summarizes anthropometrics and blood pressure, in which men showed significantly higher weight (p = 0.001), height (p = 0.001), BMI (p = 0.015), waist circumference (p = 0.001), systolic (p = 0.001) and diastolic (p = 0.001) blood pressure, and lower total PA and body fat (both p = 0.001) than women. There were no significant differences by sex in BMI percentile (p = 0.073).

**Table 1:** Demographic data of adolescents from Northwest México, n (%)

Variable	Total n = 230	Female n = 102	Male n = 128	*p* value ^*a*^
Age (years) mean±SD	18.5**±**0.4	18.4**±**0.4	18.4**±**0.3	0.247 ^b^
Socioeconomic level in US dollars per year, n (%) $0-$4080 $4081-$6935 $6936-$21,000	107 (46.9) 76 (32.8) 46 (20) 1 (0.4)	55 (53.9) 28 (27.5) 18 (17.6) 1 (1)	52 (40.6) 48 (37.5) 28 (21.8) -	0.130
Work, n (%) Yes No	48 (20.1) 182 (79.8)	12 (11.7) 90 (88.2)	36 (28.1) 92 (71.9)	0.002*
Family background, n (%) Cardiovascular diseases	25 (10.9)	10 (9.8)	15 (11.7)	0.215
Diabetes mellitus	27 (11.4)	10 (9.8)	17 (12.5)	0.662
Hypertension	49 (21.4)	20 (19.6)	29 (22.7)	0.315
Obesity	23 (10)	11 (10.7)	12 (9.4)	0.125
High cholesterol	33 (14.4)	15 (14.7)	18 (14.1)	0.190
High triglycerides	95 (41.6)	42 (44.2)	53 (55.8)	0.001*
Sports practice, n (%) Yes No	94 (40.9) 136 (59.1)	30 (29.4) 72 (70.6)	66 (51.6) 64 (50)	0.001*
Smoking, n (%) Yes No	21 (9.1) 209 (90.9)	7 (6.9) 95 (93.1)	14 (10.9) 114 (89.1)	0.287

^a^ Chi-square test

^b^ Independent T-test

* Denotes statistical significance, p < 0.05

**Table 2:** Anthropometrics, physical activity and blood pressure of adolescents from Northwest México

Variable	Total n = 230 mean ± SD	Female n = 102 mean ± SD)	Male n = 128 mean ± SD	*p* value ^*a*^
Weight (kg)	67.9 **±** 16.5	60 **±** 15.3	74.1 **±** 14.7	0.001*
Height (m)	1.7 **±** 0.1	1.6 **±** 0.6	1.7 **±** 0.6	0.001*
BMI (kg/m^2^) ^b^	23.9 **±** 4.8	23.3 **±** 5.5	24.3 **±** 4.1	0.015*
BMI percentile ^c^	59.2 **±** 30.8	55.6 **±** 31.1	62 **±** 30.4	0.073
Waist (cm)	77.3 **±** 12.5	72.3 **±** 12.5	81.1 **±** 11.1	0.001*
Body fat (%)	21.5 **±** 7.6	25.6 **±** 6.6	18.1 **±** 6.6	0.001*
Total physical activity (METs/week)	2167.4 **±** 1876.2	2515.9 **±** 1885.5	1739.5 **±** 1782.3	0.002*
Blood pressure (mmHg) Systolic Diastolic	113 **±** 11.6 71.9 **±** 9.4	107.2 **±** 10.2 69 **±** 9.1	117.6 **±** 10.5 74 **±** 9.0	0.001* 0.001*

BMI, body mass index; METs, metabolic equivalents

^a^ Independent T-test

^b^ Inverse transformation for statistical analysis; data is shown in the original units

^c^ Square-root transformation for statistical analysis; data is shown in the original units

* Denotes statistical significance, p < 0.05

### Dietary intake characteristics

Table 3 exhibits food group and nutrient intakes, with most men intakes being non-significantly higher than woman intakes (p > 0.05), except for nuts and seeds (1.1 ± 0.6 vs. 0.9 ± 0.6 oz/week, p = 0.042) and processed meats (749.8 ± 639 vs. 503.6 ± 300.3 g/week, p = 0.002), for men and women, respectively. Only fish and shellfish means were within the AHA recommended intake for men and women (513.1 ± 450.7 vs. 501.7 ± 428 g/week respectively, p = 0.671), the rest of food groups and nutrients exhibited mean values that did not reach the AHA recommendations.

**Table 3:** Food group and nutrient intakes of adolescents from Northwest México

Food group/nutrient	Total n = 228 mean (SD)	Female n = 102 mean (SD)	Male n = 126 mean (SD)	*p* value ^*a*^
F&V (cups/d)	3.60 **±** 2.2	3.34 **±** 0.7	3.29 **±** 0.7	0.772
Fish and shellfish (g/week) ^b^	507.4 **±** 422.9	501.7 **±** 428	513.1 **±** 450.7	0.671
Sodium (mg/d) ^b^	2248.3 **±** 1720.1	1994 **±** 1717.3	2458.5 **±** 1836.3	0.239
SSBs (mL/week)	120 **±** 68.1	110.2 **±** 67.0	128.1 **±** 68.3	0.52
Whole grains (oz/d)	2.6 **±** 1.5	2.6 **±** 1.4	2.7 **±** 1.5	0.49
Nuts and seeds (oz/week) ^b^	1.0 **±** 0.6	0.9 **±** 0.6	1.1 **±** 0.6	0.042*
Legumes (cups/week) ^b^	1.06 **±** 0.73	0.99 **±** 0.71	1.12 **±** 0.75	0.133
Nuts, seeds and legumes (servings/week) ^b^	1.21 **±** 0.71	1.13 **±** 0.71	1.28 **±** 0.70	0.127
Processed meats (g/week) ^b^	638.4 **±** 527.6	503.6 **±** 300.3	749.8 **±** 639	0.002*
Saturated fat (kcal/d)	157.5 **±** 61.8	153.4 **±** 63.2	160.9 **±** 60.7	0.369
Saturated fat (%/d) ^c^	10 **±** 4.6	10.6 **±** 4.8	9.5 **±** 4.4	0.129

F&V, fruits and vegetables; SSBs, sugar-sweetened beverages

^a^ Independent T test

^b^ Square-root transformation for statistical analysis; data is shown in the original units

^c^ Adjusted for energy kcal/d

* Denotes statistical significance, p < 0.05

### Cardiovascular Metrics

Classification of participants by CV metrics is shown in Table 4. The ideal level of metrics was reached by most participants for BMI percentile (70.9%), smoking (87%), systolic blood pressure (65.2%), and diastolic blood pressure (69.1%). However, approximately 30% of participants were categorized as either intermediate or poor in BMI percentile and blood pressure metrics. When focusing on PA, most of the participants were classified as intermediate (43.9%) and poor categories (30.3%). According to diet scores, most participants were classified as intermediate, with 70.4% and 84.8% for primary and secondary diet scores, respectively. Comparisons by sex showed a significantly higher prevalence of ideal blood pressure for women (p = 0.001). Ideal and intermediate levels for PA were more prevalent among men (p = 0.005), suggesting that men perform more minutes of moderate or vigorous activities every day than women. Concerning diet score, significant differences by sex were only seen in the primary diet score, with a higher prevalence in ideal level for women and a higher prevalence of intermediate level for men (p = 0.022).

**Table 4:** Classification of adolescents according to the AHA behavior metrics

Health metrics	Total n (%)	Female n (%)	Male n (%)	*p* value ^a^
BMI percentile				0.290
Ideal, n (%)	163 (70.9)	77 (76.0)	85 (66.7)	
Intermediate, n (%)	44 (19.1)	17 (16.3)	28 (21.4)	
Poor, n (%)	23 (10.0)	8 (7.7)	15 (11.9)	
Smoking				0.161
Ideal, n (%)	200 (87.0)	92 (90.4)	108 (84.1)	
Poor, n (%)	30 (13.0)	10 (9.6)	20 (15.9)	
Systolic blood pressure				0.001*
Ideal, n (%)	150 (65.2)	86 (84.6)	63 (49.2)	
Intermediate, n (%)	57 (24.8)	14 (13.5)	44 (34.1)	
Poor, n (%)	23 (10.0)	2 (1.9)	21 (16.7)	
Diastolic blood pressure				0.01*
Ideal, n (%)	159 (69.1)	80 (78.8)	78 (61.1)	
Intermediate, n (%)	49 (21.3)	17 (16.3)	33 (25.4)	
Poor, n (%)	22 (9.6)	5 (4.8)	17 (13.5)	
Physical activity				0.005*
Ideal, n (%)	59 (25.9)	20 (19.4)	40 (31.2)	
Intermediate, n (%)	101 (43.9)	40 (39.8)	60 (47.2)	
Poor, n (%)	70 (30.3)	42 (40.8)	28 (21.6)	
Primary diet score				0.022*
Ideal, n (%)	64 (27.8)	36 (35.6)	27 (21.4)	
Intermediate, n (%)	162 (70.4)	63 (61.5)	100 (77.8)	
Poor, n (%)	4 (1.7)	3 (2.9)	1 (0.8)	
Secondary diet score				0.684
Ideal, n (%)	28 (12.2)	14 (13.3)	14 (11.1)	
Intermediate, n (%)	195 (84.8)	84 (82.7)	111 (86.5)	
Poor, n (%)	7 (3.0)	4 (3.8)	3 (2.4)	

AHA, American Heart Association; BMI, body mass index

^a^ Chi-square test

* Denotes statistical significance, p < 0.05

### Level for individual Food Group and Nutrient Intakes according to the AHA criteria

**Fig. 2:**
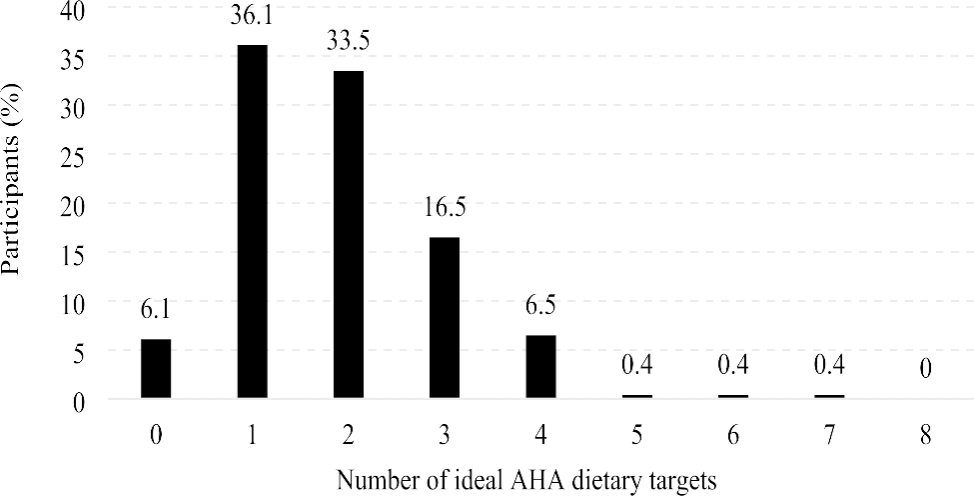
Participants (%) by number of ideal levels for dietary intake (n = 228)

According to the AHA dietary targets, Table 5 summarizes the proportion of participants by level (poor, intermediate, or ideal) for individual food groups and nutrients. Except for fish and shellfish, with 87.8% of participants reaching the ideal level (p > 0.05), most adolescents did not achieve the ideal level for the rest of the food groups and nutrients intake, with a range from 4.8% for processed meats to 37% for sodium (all p > 0.05). Significant differences in prevalence were shown only for SSBs (p = 0.013), with 10% in the ideal level. Concerning dietary metrics, the mean score ranged from 4.5 ± 3.7 for nuts, seeds, and legumes, to 9.2 ± 2.2 for fish and shellfish. Figure 2 displays the percentage of participants that achieved cero to eight ideal dietary targets, in which 36.1% and 33.5% of participants reached only one and two ideal dietary targets, respectively. Very low prevalence for ideal level in five to seven food groups and nutrients was observed, with no participants reaching the ideal level for the eight food groups and nutrients.

**Table 5:** Classification of adolescents according to the food and nutrient intakes using the AHA criteria (n = 228)

Food group	Ideal level	Intermediate + poor level	*p* value ^*a*^	Score mean ± SD ^b^
Primary Diet Score				
F&V, n (%)	67 (29.6)	161 (70.4)	0.345	6.7 **±** 2.9
Fish and shellfish, n (%)	200 (87.8)	28 (12.2)	0.281	9.2 **±** 2.2
Sodium, n (%)	84 (37.0)	144 (63.0)	0.210	7.1 **±** 3.5
SSBs, n (%)	23 (10.0)	205 (90.0)	0.013*	5.3 **±** 3.4
Whole grains, n (%)	78 (34.3)	150 (65.7)	0.061	7.1 **±** 2.8
Secondary Diet Score				
Nuts, seeds and legumes, n (%)	44 (19.1)	184 (80.9)	0.329	4.5 **±** 3.7
Processed meats, n (%)	11 (4.8)	217 (95.2)	0.208	5.2 **±** 3.0
Saturated fat, n (%) ^c^	62 (27.0)	166 (73.0)	0.544	6.1 **±** 3.7

AHA, American Heart Association; F&V, fruits, and vegetables; SSBs, sugar-sweetened beverages

^a^ Independent T test

^b^ 0 – lowest; 10 – highest

^c^ Adjusted for energy kcal/d

* Denotes statistical significance, p < 0.05

## Discussion

Due to the early development of CVD, adolescents represent a vulnerable group, especially if they are regularly exposed to unhealthy lifestyles [26]. Using AHA behavior metrics we estimated the prevalence of poor, intermediate, and ideal levels in Northwest Mexican adolescents aged 17–20 years, and when analyzed by sex, a more significant proportion of women, compared to men, exhibited ideal levels of BMI, non-smoking, and blood pressure. However, only 35.6% and 21.4% of women and men reached the ideal level in primary dietary metrics; 13.3% and 11.1% in secondary dietary metrics; and 19.4% and 31.2% in PA, respectively. Our findings are of special concern since sports activities, attitudes to sport and BMI at young ages are the most powerful predictors of adult biological risk factors, as reported by Barnekow-Bergkvist et al. [27]. This means that encouraging an ideal level of the CV behavior metrics proposed by AHA at these young ages might promote retaining these habits in adulthood.

The low prevalence of ideal levels of dietary and PA behaviors in the present study are comparable to results from 2019 in the Youth Risk Behavior Survey. According to this US survey, adolescents’ dietary and PA behaviors have not improved during the last ten years and, in some instances, have worsened. The survey showed that just a few adolescents met U.S. government recommendations for dietary or PA behaviors, and disparities by sex and race/ethnicity were found [28]. Similarly, findings from the present study indicated a sex disparity in our participants. Male students had significantly poorer dietary habits, higher blood pressure, and higher BMI but better PA behaviors than female students.

Regarding dietary behaviors in the US survey [28], trend data from 2009 to 2019 indicated no improvements in fruit or vegetable consumption, where consumption remained low. When analyzed by sex, male students exhibited a greater risk for unhealthy dietary behaviors, with a higher percentage of male than female students reporting drinking sugar-sweetened soda or pop ≥1 time/day, and an increased percentage of male students not eating or drinking enough fruit (fruit or 100% fruit juices < 1 time/day) [29]. Although dietary indicators from the US survey were not the same as in our study, results regarding differences by sex were comparable to our findings in which harmful food groups and nutrient intakes such as sodium, SSBs, and processed meats were higher in male than female students. Higher SSB intake among males compared to females has also been reported by Bleich et al. [29].

In the most recent National Health and Nutrition Survey 2021 from Mexico, for the age group of 12–19 y only one out of three adolescents consumed fruits and eggs, and only one out of four consumed vegetables and legumes daily. On the contrary, nine out of ten adolescents consumed SSBs. Sweet snacks and desserts, sweet cereals and fast food presented similar percentages of consumers as dairy; situation that is neither desirable nor compatible with an adequate state of health [9]. These numbers are similar than our results in the present study with adolescents, regarding F&V and SSBs intake.

Considering PA levels, the US Survey among adolescents [28] detected that the overall prevalence of health-promoting PA behaviors in 2019 was low. The proportion of students meeting the guidelines decreased, and a higher percentage of males than females met the recommendations across all the PA behaviors. In México, some studies have reported poor levels in 31.9% of high school adolescents and different proportions of ideal levels in males (52.6%) and females (30.5%) [30]. Other authors highlighted that some specific activities, such as active commuting to school, are every day in youth and reported by 70.8% of adolescents [31]. These studies are critical because they suggest that adolescents can improve their PA levels by including some activities that are part of their daily routine. In the present study, the prevalence of ideal level of PA are similar to the prevalence reported in other studies, such as in adolescents from low-to-middle income countries (15.4%) [32] and Chinese youth (16.6%) [33]. High PA levels are associated with a reduced risk of developing overweight, obesity, diabetes mellitus, and elevated blood pressure, all components of CVH [34]. In addition, previous research has established that strategies to treat and prevent overweight and obesity should include promoting PA and increases in cardiorespiratory fitness in adolescents [35].

Healthy eating and healthy PA during early ages are critical for optimal growth and development, academic outcomes, and prevention of chronic diseases, including T2D, heart disease, hypertension, and obesity [36]. On the contrary, inadequate intake of food groups and nutrients recommended for CVH is of particular concern since poor diets, and lack of moderate and/or vigorous PA increase the risk for obesity and related health problems in adulthood. Moreover, different developmental trajectories of unhealthy behaviors such as smoking, sedentary life, lack of or low fruit intake, and drunkenness have been identified in adolescents [36]. The most severe trajectory is for those with higher levels of unhealthy behaviors over time. This is particularly important since behaviors throughout adolescence may remain into adulthood, and unhealthy factors such as smoking and alcohol abuse may become persistent habits and potential addictions in the future. Besides, the sustained practice of ideal CV behaviors is associated with a significant decrease in the CVD risk [37]. High levels of CVH in late adolescence or young adulthood were associated with very low rates of premature CVD and mortality over 32 years of follow-up in a study by Perak et al. [38]. Therefore, early adoption of habits related to CVH involving a healthy diet and adequate PA level during adolescence is essential to prevent the development of atherosclerosis and CVD [37].

In México, overweight and obesity are critical health challenges for adults and adolescents since this group has been marked by a significant increasing trend in its combined prevalence, with 42.9% in 2021. The highest prevalence of obesity in men adolescents was reported in North-Pacific México (32%) [9]. In the present study, 29.1% of the participants did not achieve the ideal level of BMI. Other studies have reported similar non-ideal levels of BMI in adolescents from Europe (23.2%) and the United States (31%) [38]. Our results are alarming compared with the low prevalence in countries like China, which have a 6.5% rate of non-ideal BMI for young people [39]. Obesity is associated with CVD, cardiometabolic diseases, and premature mortality in adulthood. Hence, the importance of early prevention strategies in this group of age. There is an urgent need for multicomponent approaches for adolescents, including nutrition needs, policy and environmental changes, opportunities to learn about (and practice) making healthy choices, and their barriers and motivations for adopting a healthier eating pattern [29].

CVD are still the leading cause of death in México, representing 24% of all deaths, with Northern México having the highest rate of annual CV events [40]. This high rate might be related to particular dietary patterns, such as the Western diet [41], common in the Northern region of México. Dietary habits in this region include high consumption of red and processed meat, white flour, saturated fat, and sugary and salty foods [42, 43].

Our results in adolescents reflect a low ideal level of secondary diet score with low consumption of nuts, seeds, and legumes and high intakes of processed meats and saturated fats. A low percentage of participants achieved the ideal level for all these food groups and nutrient intakes. Therefore, it is vital to monitor CVH-related dietary factors during childhood and adolescence and promote the recommended intakes of protective- and reductions in harmful-food groups and nutrients [44].

None of the participants achieved the total eight ideal AHA dietary targets, and 6.1% reached cero ideal dietary targets (Fig. 2). Other studies have shown similar results of low achievement for AHA dietary targets in adolescents [45–47]. Less than a third of participants reported an ideal level in the primary diet score (Table 4). Almost a third of participants (29.6%) achieved higher ideal levels of F&V (Table 5), which is a higher prevalence than in US adolescents [48] and European adolescents [45]. Fish and shellfish was the food group in which most participants achieved ideal levels (87.8%), which was substantially higher than that reported by US adolescents (29.7%) and Europeans (22.3%) [45, 47]. Fish and shellfish are foods that are frequently consumed in this region of Mexico, therefore they are part of the Northwest Mexican diet. This is because this food group is available and affordable due to the proximity of our region to the coast. They are also typically consumed as easy-to-prepare cold dishes during summer months, in which the weather is very hot and sunny, plus the summer lasts longer than in other regions of Mexico. Regarding SSBs, participants achieved the lowest prevalence at the ideal level (10%), which was markedly lower compared to European adolescents (51.7%) [47], and US adolescents (women: 31.2%, men: 20.6%), but similar to other low-and middle-income countries [46]. Consumption of SSBs has been linked to CVD development [49], mainly through mechanisms including obesity and diabetes. The Mexican government has taken actions to help reduce SSB intake, like the tax campaign in 2014 that implemented a 10% tax on SSBs as part of a multifaceted strategy. Results have shown a gradual reduction in SSB intake [50]. Previous studies have given a possible explanation for the high intake of SSB in some specific race/ethnic populations since beverage companies disproportionately market SSBs to Hispanic youths [51].

### Strengths and limitations

The main strength of our study is the assessment of CV health metrics in an undeserved group, which is vulnerable to adopting early unhealthy behaviors that might increase their CV risk in the future. Dietary information was collected using a semi-structured FFQ specifically designed to assess CVH-related foods and nutrients according to AHA dietary targets and validated previously for its use in this population [19]. Another strength of our study is the administration of the questionnaires directly to adolescents (and not to their parents) and the three-month interval re-administration, which may have led to good reproducibility results. The sample had an equitable distribution of male and female participants, and the sample size was relatively large compared to other studies in adolescents [52]. The present study provides valuable information on CVH in a sample of Northern Mexican adolescents. To the best of our knowledge, there are no studies reporting CV metrics according to AHA in adolescents from this region.

An important limitation of this study includes the lack of measurement of blood metrics (blood glucose and total cholesterol) to fully assess CVH AHA scores [6]. These blood metrics were not collected due to a limited budget. Another limitation worth considering is the use of questionnaires to assess PA and diet, which rely on participant memory and may not reflect the usual habits [53]. Although our validated FFQ and nutrient database likely comprehensively assessed typical intake, other nutrients or foods were not included here (e.g., phytochemicals) that may influence CVH. Another limitation is that the present study only included freshman students, who may be different from other groups of adolescents. For some individuals, the “freshmen transition” is a transformational experience during the critical period of leaving high school for university entry. For a successful transition, students need to maintain positive attitudes in aspects such as relationships, individual life philosophy, self-identity, and maintaining physical and mental health, all of which could potentially affect survey and questionnaire responses [54]. Moreover, their new food choices differ because of the need for adapting to new class schedules, exposure to nearby fast-food establishments, new social relationships, personal tastes, and individual budget [55].

Being students in a university facility, our participants have access to sport-related infrastructure for different sports or disciplines (i.e., gym, swimming, tennis, taekwondo, basketball, etc.). They are encouraged to take credits in an institutional health program. These facilities are not often available in some other colleges or accessible to non-student adolescents [34]. Community programs, including sports facilities, could be great value strategies if adopted in different educational centers or open to other community members. Despite these limitations, our study establishes a precedent by evaluating CV behavior metrics according to the AHA criteria in a neglected group due to their early age.

The present study provides an integrative assessment of the CV risk of college adolescents from a geographical area that is national and internationally recognized for its high prevalence of overweight and obesity, and for the known changes in their dietary and PA patterns. These changes are partly due to their Westernization process and its economic development for being in a border area adjacent to a developed country, and many other associated factors. In addition, changes occurring in adolescence and during the school transition lie on this age-group, which together with the food environment make them vulnerable to cardiometabolic diseases at an early age. As far as we know, this study is one of the fewer on Northern Mexican adolescents that considers the AHA criteria as comparison parameters with college students from other countries. Our results can be the guideline to strengthen public health policies within this population.

## Conclusion

Due to the low prevalence of ideal levels in CV behavior metrics, adolescents from Northwest México represent a high-risk group for developing long-term unhealthy habits. Moreover, their diet and PA characteristics make them vulnerable to CVD complications early in adulthood. Multi approach programs and targeted community-based interventions based on the identified least prevalent ideal metrics are needed for expeditious health promotion and CVD prevention. Interventions focused on the AHA guidelines, incorporating traditional dishes, and considering adolescent emotional transitions may be relevant for public health in this and similar populations. Finally, public policies should be focused on increasing moderate and/or vigorous PA and promoting a heart-healthy diet, weight control, and avoiding smoking, among others, as behaviors strongly associated with long-term CVH.

## Electronic supplementary material

Below is the link to the electronic supplementary material.

Supplementary Material 1

Supplementary Material 2

## Data Availability

The datasets generated and analyzed during the current study are not publicly available, but are available from the corresponding author on a reasonable request through an e-mail at ana.renteria@itson.edu.mx.

## Abbreviations

AHA: American Heart Association
BMI: Body Mass Index
CV: Cardiovascular
CVD: Cardiovascular diseases
CVH: Cardiovascular health
FFQ: Food Frequency Questionnaire
F&V: Fruits and vegetables
IPAQ: International Physical Activity Questionnaire
MET: Metabolic equivalent of task
PA: Physical activity
SD: Standard deviation
SSBs: Sugar-sweetened beverages
T2D: Type 2 diabetes
