# Supplementary material for: Low prevalence of ideal levels in cardiovascular behavior metrics among Mexican adolescents
Source: BMC Public Health. 2023 Jun 12;23:1125. doi: 10.1186/s12889-023-15959-3 (PMC10259807; doi:10.1186/s12889-023-15959-3)
Supplement: Supplementary file 1 — Supplementary Material 1 [file 12889_2023_15959_MOESM1_ESM.docx]

**Additional File 1.** Definitions of ideal, intermediate, and poor categories for BMI, physical activity, smoking, and blood pressure for children and adolescents 12-19 years according to the AHA definition of cardiovascular health (Virani SS, Alonso A, Benjamin EJ, Bittencourt MS, Callaway CW, Carson AP, et al. Heart Disease and Stroke Statistics-2020 Update: A Report From the American Heart Association. Circulation. 2020;141:E139–596. https://doi.org/: 10.1161/CIR.0000000000000757.).

| Metric | Ideal | Intermediate | Poor |
| --- | --- | --- | --- |
| Body mass index (percentile) | <85^th^ | 85^th^– 95^th^ | > 95^th^ |
| Physical activity | ≥60 min of moderate or vigorous every day | >0 and <60 min of moderate or vigorous every day | None |
| Smoking | Never tried, never smoked whole cigarette | - | Tried during the prior 30 d |
| Blood pressure (percentile) | < 90^th^ or SBP < 120 mmHg or DBP < 80 mmHg | 90^th^– 95^th^ or SBP 120-129 mmHg or DBP <80 mmHg | > 95^th^ or SBP ≥ 130 mmHg or DBP ≥ 80 mmHg |

AHA, American Heart Association; SBP, systolic blood pressure, DBP, diastolic blood pressure

Source: Modified from Lloyd-Jones DM, Hong Y, Labarthe D, Mozaffarian D, Appel LJ, Van Horn L, et al. Defining and setting national goals for cardiovascular health promotion and disease reduction: The American Heart Association’s strategic impact goal through 2020 and beyond. Circulation. 2010;121:586–613.
