# Supplementary material for: Low prevalence of ideal levels in cardiovascular behavior metrics among Mexican adolescents
Source: BMC Public Health. 2023 Jun 12;23:1125. doi: 10.1186/s12889-023-15959-3 (PMC10259807; doi:10.1186/s12889-023-15959-3)
Supplement: Supplementary file 2 — Supplementary Material 2 [file 12889_2023_15959_MOESM2_ESM.docx]

**Additional File 2.** AHA dietary targets and healthy diet score for defining cardiovascular health (Virani SS, Alonso A, Benjamin EJ, Bittencourt MS, Callaway CW, Carson AP, et al. Heart Disease and Stroke Statistics-2020 Update: A Report from the American Heart Association. Circulation. 2020;141:E139–596. https://doi.org/: 10.1161/CIR.0000000000000757).

| Dietary metrics | AHA Target ^a^ | Alternative Scoring  Range ^a^ |
| --- | --- | --- |
| Primary dietary metrics |  |  |
| F&V | 0 to ≥ 4.5 cups/d ^b^ | 0-10 |
| Fish and shellfish | 0 to ≥ 200 g/week | 0-10 |
| Sodium | ≤ 1500 to >4500 mg/d | 10-0 |
| SSBs | ≤ 36 to >210 fl oz/week | 10-0 |
| Whole grains | 0 to ≥ 3 oz/d | 0-10 |
| Secondary dietary metrics |  |  |
| Nuts, seeds and legumes | 0 to ≥ 4 servings/week (nuts/seeds=1 oz; legumes ½ cup) | 0-10 |
| Processed meats | ≤100 to >500 g/week | 10-0 |
| Saturated fat | ≤ 7 to >15 (% energy/d) | 10-0 |

AHA, American Heart Association; F&V, fruits and vegetables; SSBs, sugar-sweetened beverages.

^a^ Consistent with other dietary pattern scores, the highest score (10) was given for meeting or exceeding the AHA target (e.g., at least 4.5 cups of fruit and vegetables per day; no more than 1500 mg/d of sodium), and the lowest score (0) was given for zero intake (protective factors) or for very high intake (harmful factors). The score for each metric was scaled continuously within this range. For harmful factors, the level of high intake that corresponded to a zero score was identified as approximately the 90th percentile distribution of US population intake (Rehm et al., 2016) and Latino adolescents (Leung et al., 2017).

^b^ Including up to one 8-oz serving per day of 100% fruit juice and up to 0.42 cups/d (3 cups/week) of starchy vegetables such as potatoes or corn.

Sources

Leung CW, Tester JM, Rimm EB, Willett WC. SNAP Participation and Diet-Sensitive Cardiometabolic Risk Factors in Adolescents. Am J Prev Med. 2017;52:S127–37. <https://doi.org/:10.1016/j.amepre.2016.06.011>.

Lloyd-Jones DM, Hong Y, Labarthe D, Mozaffarian D, Appel LJ, Van Horn L, et al. Defining and setting national goals for cardiovascular health promotion and disease reduction: The american heart association’s strategic impact goal through 2020 and beyond. Circulation. 2010;121:586–613.

Rehm CD, Peñalvo JL, Afshin A, Mozaffarian D. Dietary Intake Among US Adults, 1999-2012. JAMA. 2016;315:2542–53. https://doi.org/: 10.1001/JAMA.2016.7491.

Virani SS, Alonso A, Benjamin EJ, Bittencourt MS, Callaway CW, Carson AP, et al. Heart Disease and Stroke Statistics-2020 Update: A Report from the American Heart Association. Circulation. 2020;141:E139–596. https://doi.org/: 10.1161/CIR.0000000000000757.
